# MRI diagnosis of preterm prelabor rupture of membranes and uterine puncture site–amniotic cavity fistula following fetoscopic laser photocoagulation

**DOI:** 10.1186/s12884-026-09100-6

**Published:** 2026-04-16

**Authors:** Yunlan Wang, Gang Ning, Yan Sun, Haibo Qu

**Affiliations:** 1 Department of Radiology, West China Second University Hospital, Key Laboratory of Birth Defects and Related Diseases of women and Children, Sichuan University, Ministry of Education, Chengdu City, Sichuan Province China; 2 Department of Radiology, Panzhihua Central Hospital, Panzhihua City, Sichuan Province China

**Keywords:** Magnetic resonance imaging, Twin-twin transfusion syndrome, Fetoscopic laser photocoagulation, Selective fetal growth restriction, preterm prelabor rupture of membranes

## Abstract

Preterm prelabor rupture of membranes (PPROM) following fetoscopic laser photocoagulation (FLP) is a rare but potentially life-threatening complication. The occurrence of PPROM with a uterine puncture site–amniotic cavity fistula and localized fluid collection in the vesicouterine pouch has not been previously reported, and its diagnosis and management present significant clinical challenges. A 27-year-old primiparous woman with monochorionic diamniotic (MCDA) twin pregnancy was diagnosed at 17 weeks of gestation with Quintero stage I twin–twin transfusion syndrome (TTTS) complicated by type I selective fetal growth restriction (sFGR), an adherent donor twin, and velamentous cord insertion. Following multidisciplinary consultation and comprehensive counseling, she underwent FLP using the SOLOMON technique at 17 weeks and 1 day of gestation. Routine postoperative ultrasound surveillance at 19 weeks and 6 days revealed a slit-like defect in the anterior uterine wall with an adjacent fluid collection, initially raising suspicion of amniotic sac prolapse. Fetal MRI at 20 weeks confirmed PPROM with uterine puncture site–amniotic cavity fistula, localized amniotic fluid accumulation in the vesicouterine pouch, and intracranial abnormalities in both fetuses—including right lateral ventriculomegaly and, in the recipient twin, focal parenchymal atrophy and abnormal signal intensity in the right cerebral hemisphere. Serial ultrasound examinations demonstrated progressive enlargement of the uterine wall defect, increasing fluid accumulation in the vesicouterine pouch, and worsening oligohydramnios. After repeated multidisciplinary discussions and thorough counseling, the patient elected termination of pregnancy. Post-induction laparoscopic exploration confirmed a healed puncture scar on the anterior uterine wall with no overlying amniotic membrane. This case highlights a rare but serious fistulous complication after FLP for stage I TTTS with sFGR. It underscores the importance of systematic postoperative imaging surveillance and the critical role of MRI in definitive diagnosis. The report also prompts reflection on the timing of intervention in borderline-indication cases and the need for individualized counseling that includes expectant management as a potential alternative.

## Introduction

TTTS affects approximately 10%–15% of MCDA twin pregnancies and results from unbalanced inter-twin blood flow through placental vascular anastomoses [1, 2]. Although FLP has substantially improved perinatal outcomes, its application in Quintero stage I TTTS complicated by sFGR remains controversial [3]. Postoperative fistulous complications in this setting are exceedingly rare, and their clinical presentation, imaging features, and optimal management have not been well characterized. We report a case of stage I TTTS with type I sFGR that developed PPROM and uterine puncture site–amniotic cavity fistula 20 days after FLP using the SOLOMON technique, and we review the relevant literature to highlight the diagnostic challenges and therapeutic considerations associated with this rare complication.

## Case report

### Patient information

A 27-year-old primiparous woman was admitted to our hospital at 16 weeks and 6 days of gestation. Three days before admission, an external ultrasound examination had revealed oligohydramnios in one sac of MCDA twin pregnancy. The patient reported abdominal distension but denied abdominal pain, vaginal bleeding, or leakage of amniotic fluid. A detailed ultrasound examination performed at our institution at 17 weeks of gestation (Table 1) established the diagnosis of Quintero stage I TTTS complicated by type I sFGR. Additional findings included an adherent donor twin and velamentous cord insertion.

**Table 1:** Ultrasound results at 17 weeks of gestation: The primary fetal biometrics

Parameter	Twin 1 (LSP)	Twin 2 (LOP)
BPD (cm)	3.71	3.25
AC (cm)	11.24	10.37
HC (cm)	12.83	12.16
EFW (g)	170 ± 25	151 ± 22
DVP (cm)	8.0	1.0
Umb. Artery S/D	5.00	5.77
Fetal heart rate	144/min, rhythm	157/min, rhythm
Umbilical cord	Nuchal cord (single loop); cord insertion at the right upper portion of the anterior uterine wall	No nuchal cord; velamentous cord insertion: cord inserts at the placental margin, with umbilical vessels traversing the membranes before reaching the placental disc
Placenta/ diaphragm	Single posterior placenta; thin inter-twin membrane visualized	
cervix	Cervix length about 4.5 cm	

### Clinical decision-making

Following a multidisciplinary team consultation, the patient received comprehensive counseling regarding all available management options, including expectant management and FLP, together with the associated risks such as preterm birth and PPROM. After detailed counseling, the patient elected to proceed with FLP.

### Surgical procedure

The procedure was performed under ultrasound guidance at 17 weeks and 1 day of gestation. A 3.9-mm operative sheath was introduced into the recipient twin’s amniotic cavity through a puncture site located inferior and left to the umbilicus. After insertion of a 3.3-mm fetoscope, warm saline was infused to optimize visualization for assessment of fetal positions, umbilical cord insertions, the inter-twin membrane, and placental vascular anatomy. Direct endoscopic inspection revealed multiple fine vascular anastomoses in the equatorial region of the placenta and two large arteriovenous connections. A 600-µm laser fiber was advanced through the working channel. Under direct vision, all visible communicating vessels were sequentially photocoagulated at 25 W. Continuous linear coagulation (SOLOMON technique) was then applied to connect the ablated areas. At the conclusion of the procedure, approximately 500 mL of amniotic fluid was slowly aspirated. Intraoperative ultrasound showed a small volume of pelvic fluid, which remained stable during a 20-minute observation period. The instruments were then withdrawn. The uterine puncture site was sutured, and hemostatic compression was applied. The total operative time was 70 min, and the procedure was completed without intraoperative complications. Postoperatively, the patient received standard prophylactic regimens, including antibiotics, tocolytics, and thromboprophylaxis.

### Postoperative course and imaging surveillance

**Fig. 1:**
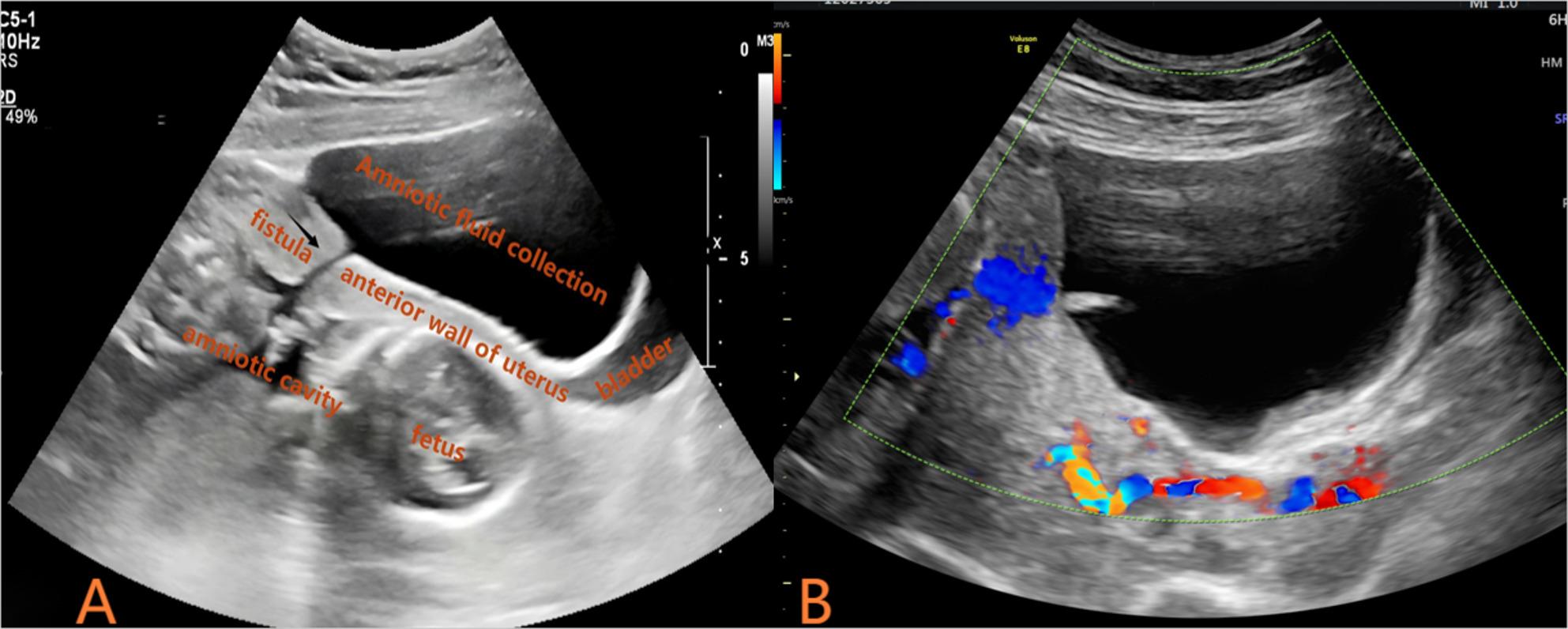
Transabdominal ultrasound at 19 weeks and 6 days of gestation. **A** sagittal view of the lower anterior uterine wall shows a slit-like defect (arrow) at the previous fetoscopic puncture site, with a large anechoic fluid collection within the vesicouterine pouch. **B** No vascular flow was detected within the collection on color Doppler imaging

**Fig. 2:**
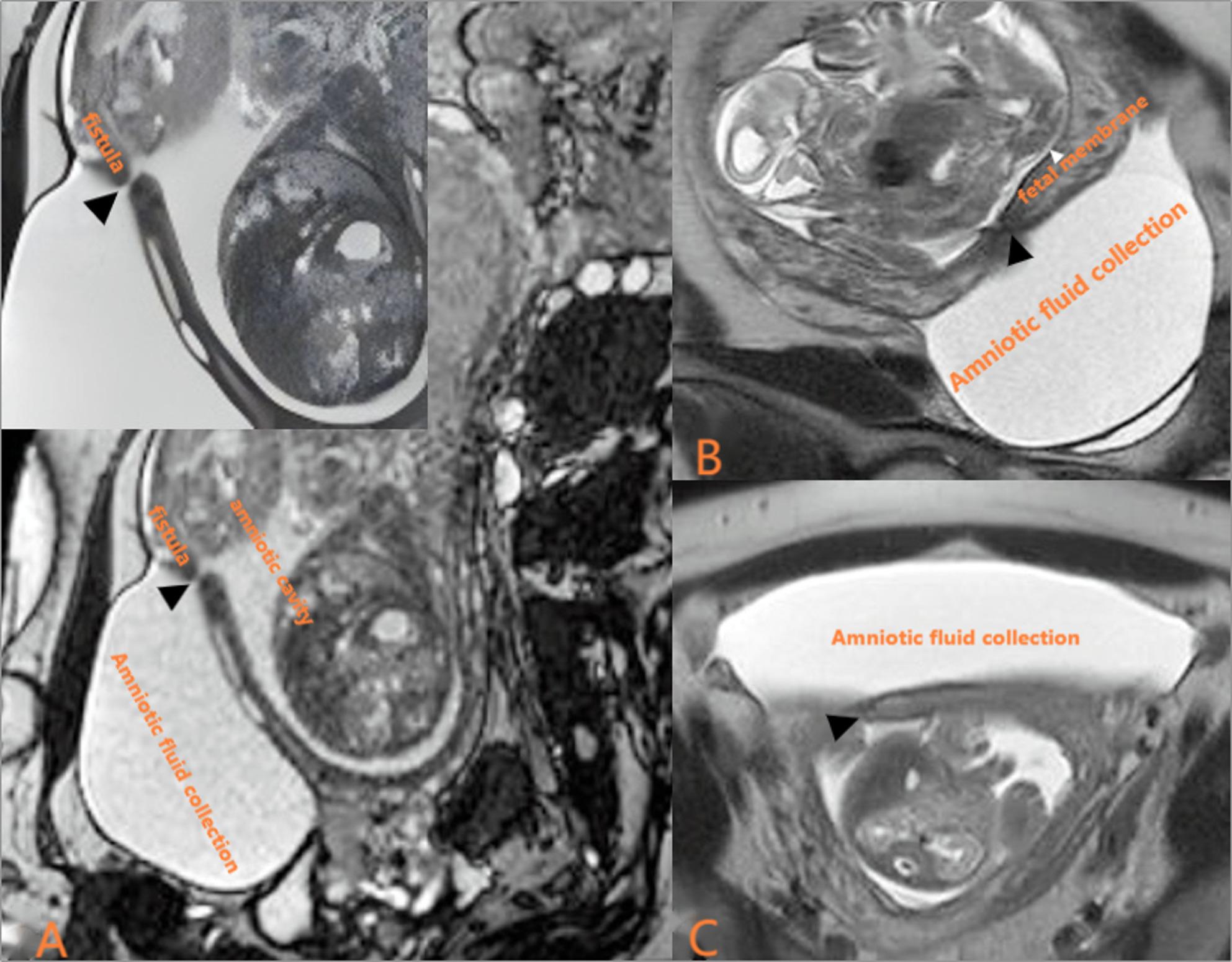
MRI at 20 weeks of gestation. **A** Oblique sagittal BSSFP image shows a fistulous tract (black arrow) through the anterior uterine wall at the previous puncture site, with localized fluid in the vesicouterine pouch compressing the bladder. Intra amniotic fluid is reduced. **B** Coronal T2WI reveals undulating, collapsed fetal membranes (white arrows). **C** Axial T2WI confirms the fistulous tract (black arrow) and the fluid collection

The patient underwent routine postoperative ultrasound monitoring. On postoperative day 20 (at 19 weeks and 6 days of gestation), an outpatient ultrasound examination revealed a slit-like defect measuring approximately 0.3 cm in transverse diameter in the lower anterior uterine wall. Anterior to this defect, within the vesicouterine pouch, a large anechoic collection measuring approximately 10.4 cm × 6.6 cm × 11.0 cm was observed. This fluid collection communicated with the uterine cavity, raising suspicion of amniotic sac herniation through the uterine puncture site (Fig. 1). Key fetal biometric parameters from this examination are presented in Table 2. Following readmission, pelvic and fetal brain magnetic resonance imaging (MRI) was performed. MRI confirmed a focal discontinuity in the lower anterior uterine wall, corresponding to the defect identified on ultrasound. The vesicouterine pouch contained a sizable fluid collection measuring approximately 5.12 cm × 8.31 cm × 11.18 cm, which appeared hypointense on T1-weighted images (T1WI) and hyperintense on T2-weighted images (T2WI). This collection communicated with the uterine cavity through the uterine wall defect. Intrauterine amniotic fluid volume was reduced, and the fetal membranes appeared collapsed with an undulating contour. These findings were consistent with PPROM and uterine puncture site–amniotic cavity fistula, resulting in localized amniotic fluid accumulation within the vesicouterine pouch (Fig. 2). Fetal MRI also identified intracranial abnormalities. Both fetuses demonstrated mild dilatation of the right lateral ventricle. In the donor twin, findings included focal parenchymal atrophy, cortical thinning, and abnormal signal intensity in the right cerebral hemisphere, indicative of established brain injury. The placenta was located on the posterior uterine wall and fundus, with velamentous cord insertion confirmed for the donor twin. Subsequent serial ultrasound examinations documented progressive enlargement of the anterior uterine wall defect, a concomitant increase in the volume of fluid within the vesicouterine pouch, and a progressive decline in intra-amniotic fluid volume.

**Table 2:** Ultrasound results at 19 + 6 weeks of gestation: key fetal biometrics

Parameter	Twin 1 (LOP)	Twin 2 (Transverse)
BPD (cm)	4.36	4.00
AC (cm)	14.47	13.51
HC (cm)	16.47	16.10
EFW (g)	308 ± 45	261 ± 38
DVP (cm)	2.9	2.0
Umb. Artery S/D	4.0	4.1

### Management and outcome 

Given the clinical complexity and the potential risks to both maternal and fetal well-being, a multidisciplinary team consultation was reconvened, including specialists in obstetrics, maternal-fetal medicine, diagnostic imaging, and fetal surgery. The patient received comprehensive counseling regarding the two available management options: continuing the pregnancy under intensive surveillance—with explicit discussion of the associated risks, including enlargement of the uterine puncture site, hemorrhage, preterm delivery, and intrauterine fetal demise—or proceeding with termination of the pregnancy. After fully understanding the anticipated risks and potential outcomes of each option, the patient and her family elected to proceed with termination. At 20 weeks and 2 days of gestation, ultrasound-guided aspiration of the fluid collection in the vesicouterine pouch was performed, followed by intra-amniotic injection of ethacridine lactate for labor induction. Vaginal delivery of two stillborn fetuses occurred two days later. To directly assess the intraabdominal findings, diagnostic laparoscopy was performed on the following day. Laparoscopic examination revealed a well-healed scar at the original uterine puncture site on the left anterior wall. The uterine serosa appeared smooth, with no evidence of adherent chorioamniotic membrane tissue. The patient’s postoperative recovery was uneventful, and she was discharged two days after the procedure.

## Discussion

Uterine puncture site–amniotic cavity fistula with localized amniotic fluid accumulation in the vesicouterine pouch following FLP is an exceedingly rare but potentially life-threatening complication. To date, no identical case has been reported in the published literature. Unlike typical PPROM, this case demonstrated an abnormal fistulous tract extending from the uterine wall puncture defect to the amniotic cavity, with fluid localized to the vesicouterine pouch rather than freely disseminating within the peritoneal cavity. This anatomical pattern differs from previously reported cases of uterine perforation or amniotic sac prolapse, in which the fetal membranes remain intact [4–7].

### Pathophysiological mechanisms

Fetal membrane integrity depends on the dynamic balance and remodeling of the extracellular matrix at the amnion–chorion interface [8]. Fetoscopic puncture disrupts the anatomical continuity of both the uterine wall and the fetal membranes, creating the substrate for fistula formation. Multiple procedure-related factors may act synergistically to impede normal healing of the puncture tract. These include earlier gestational age at intervention (when tissues are thinner), prolonged operative time, higher cumulative laser energy, and more extensive coagulation zones required for complex placental vascular anastomoses—such as those treated with the SOLOMON technique [9]. Excessive distension of the fetal membranes associated with polyhydramnios in the recipient twin may further increase membrane fragility [10, 11]. The combined effect of these factors can prevent healing of the uterine wall defect and the overlying membrane disruption, ultimately resulting in an abnormal fistulous communication between the uterine puncture site and the amniotic cavity.

### Diagnostic considerations

Ultrasound remains the preferred initial modality for postoperative surveillance after fetoscopic surgery [12]. In this case, ultrasound detected the uterine wall defect and adjacent fluid collection but could not definitively characterize their nature. MRI provided conclusive diagnostic evidence by directly demonstrating the fistulous tract, the interruption of the amniotic sac at the defect site, and the localized fluid accumulation in the vesicouterine pouch. The progressive reduction in intrauterine amniotic fluid volume concomitant with increasing localized fluid in the vesicouterine pouch—a “separation” phenomenon—further corroborated the diagnosis. The principal diagnostic advantages of MRI in this context include its ability to delineate fistulous tracts with high soft‑tissue contrast, differentiate fistula from amniotic sac prolapse, comprehensively assess fetal intracranial structures and placental morphology, and identify secondary changes such as infection or hemorrhage using functional sequences like diffusion‑weighted imaging(DWI) [13]. These capabilities make MRI indispensable for definitive diagnosis when ultrasound findings are equivocal.

###  Reflections on clinical decision-making

The indications and optimal timing for FLP in Quintero stage I TTTS complicated by sFGR remain debated [14, 15]. International consensus generally recommends intervention from stage II onward, while considering expectant management a reasonable option for asymptomatic stage I cases [3]. The present case involved stage I TTTS with type I sFGR, characterized by severe oligohydramnios in the donor twin and an adherent placental relationship—features associated with increased risk of disease progression. Following multidisciplinary discussion, the team elected to perform FLP using the SOLOMON technique, with the goals of preventing donor twin demise and improving the intrauterine environment for the growth-restricted fetus. This intervention represented an active clinical decision that inherently carried surgical risks. The occurrence of this complication may be related to the early gestational age at intervention—when the biomechanical properties and healing capacity of the myometrium and fetal membranes differ—and to the more extensive surgical approach inherent to the SOLOMON technique [11, 16]. From a retrospective standpoint, expectant management also constituted a reasonable clinical option in this instance. Although the natural disease course under conservative observation cannot be known with certainty, such an approach might theoretically have avoided this iatrogenic complication. This reflection does not invalidate the clinical rationale underlying the initial decision; rather, it highlights the inherent difficulty of establishing definitive intervention thresholds in borderline cases.

### Clinical implications and limitations 

This case offers several important lessons. First, it underscores the need for systematic postoperative imaging surveillance after FLP, with specific attention to the uterine puncture site, the vesicouterine pouch, and amniotic fluid volume. Second, it demonstrates the critical role of MRI when ultrasound findings are suspicious or inconclusive. Third, it highlights the complexity of decision-making in stage I TTTS with sFGR, emphasizing that no single management pathway is universally correct and that individualized counseling must include expectant management as a potential alternative.

The principal limitation of this report is its single-case nature, which precludes generalization of the findings. Additionally, the retrospective analysis is subject to inherent biases. Nevertheless, the rarity of this complication and the detailed imaging and clinical data presented provide valuable insights for clinicians involved in fetal therapy and postoperative care.

## Conclusions

This case highlights that fistulous complications after fetoscopic surgery, though rare, require systematic imaging surveillance for timely diagnosis. MRI is essential when ultrasound findings are equivocal. The report also underscores the need for individualized decision-making in borderline-indication cases, where expectant management may be a reasonable alternative to intervention.

## Data Availability

The datasets obtained and/or analyzed during the current study are available from the corresponding author on reasonable request.
